# Rotational Dynamics of Optically Trapped Human Spermatozoa

**DOI:** 10.1155/2014/154367

**Published:** 2014-01-29

**Authors:** Elavarasan Subramani, Himanish Basu, Shyam Thangaraju, Sucheta Dandekar, Deepak Mathur, Koel Chaudhury

**Affiliations:** ^1^School of Medical Science and Technology, Indian Institute of Technology Kharagpur, Kharagpur, West Bengal 721 302, India; ^2^Tata Institute of Fundamental Research, Homi Bhabha Road, Mumbai 400 005, India; ^3^Department of Biochemistry, Seth GS Medical College and KEM Hospital, Mumbai 400 012, India

## Abstract

*Introduction*. Optical trapping is a laser-based method for probing the physiological and mechanical properties of cells in a noninvasive manner. As sperm motility is an important criterion for assessing the male fertility potential, this technique is used to study sperm cell motility behavior and rotational dynamics. *Methods and Patients*. An integrated optical system with near-infrared laser beam has been used to analyze rotational dynamics of live sperm cells from oligozoospermic and asthenozoospermic cases and compared with controls. *Results*. The linear, translational motion of the sperm is converted into rotational motion on being optically trapped, without causing any adverse effect on spermatozoa. The rotational speed of sperm cells from infertile men is observed to be significantly less as compared to controls. *Conclusions*. Distinguishing normal and abnormal sperm cells on the basis of beat frequency above 5.6 Hz may be an important step in modern reproductive biology to sort and select good quality spermatozoa. The application of laser-assisted technique in biology has the potential to be a valuable tool for assessment of sperm fertilization capacity for improving assisted reproductive technology.

## 1. Introduction

An optical tweezer (optical trap) is a biophotonic device that uses the momentum generated by a sharp spatial gradient of laser intensity to trap micron-sized particles. This physical phenomenon was first described by Ashkin more than four decades ago [1], and the technique was first used for trapping live cells twenty-five years ago [2]. The sharp spatial intensity gradient is accomplished by means of a microscope objective (usually 100X) with high numerical aperture, and the trapping strength that can be achieved depends upon the optical geometry of the trap and the object that is sought to be trapped [3]. Optical tweezers are increasingly being used as a noninvasive method to study the mechanical properties, behavior, and physiology of cells and also to spatially manipulate single cells [4–6]. Characterization of the dynamics of the rotational motion of flagellar mutants in *Chlamydomonas reinhardtii* is one recent example of the utility of this technique [7] for studying live cells kept under physiological conditions.

Study of spermatozoa dynamics, specifically the generation of motility forces, has been a subject of contemporary interest [8–11]. Optical traps have been used to probe the force generated by motile spermatozoa and their variations in abnormal sperm cells [12–14]. More recently, laser tweezers have been used in combination with computer tracking software and robotic technology for high-throughput sorting and measurement of sperm characteristics [15–19]. Measurement of sperm motility and swimming force has generated considerable interest amongst clinical researchers, as these parameters are useful for determining fertilization rates. A correlation has been observed between sperm swimming force and swimming speed [20]. Such relationships between swimming forces and swimming speeds of primate species, including chimpanzee, rhesus macaque, human, and gorilla, have also been used to deduce the evolutionary patterns of sperm in relation to mating patterns of different primates [21].

Classically optical trapping has been carried out using near-infrared (nIR) lasers, with spectral ranges of 700–1200 nm, as these wavelengths are only weakly absorbed by live cells [22, 23]. Although this spectral range is assumed to be safe and noninvasive to the cells, there are concerns that the laser light, *per se,* might have damaging effects on the certain types of living specimens, such as DNA damage, loss of membrane potential, cloning efficiency reduction, and loss of viability [24–27].

Although various properties of spermatozoa including swimming speed and swimming force before and after optical trapping have, to a certain extent, been studied in different mammalian species, no reports exist on the study of rotational dynamics of a single live sperm cell within a trap. In the present study, we report real-time changes in the rotational dynamics of optically trapped, abnormal human spermatozoa, including oligozoospermic and asthenozoospermic cells, as compared to normal sperm cells. Furthermore, their progressive motility before and after trapping is systematically investigated.

## 2. Materials and Methods

### 2.1. Subject Selection and Sperm Collection

Asthenozoospermic (*n* = 24) and oligozoospermic (*n* = 22) semen samples were randomly collected from subjects (28–40 years) reporting at the King Edward Memorial (KEM) Hospital, Mumbai, for male factor infertility. The control group consisted of proven fertile men (*n* = 20) whose partners had delivered healthy babies during the last six months without assisted reproductive technology (ART). Semen ejaculates were collected after three to five days of sexual abstinence in sterile containers and were examined in the laboratory within 1 h as per World Health Organization (WHO) 2010 guidelines [28]. Fifty sperm cells from each sample were optically trapped for analysis. Institutional approval was acquired from the Research Ethics Committee to carry out this study and written informed consent was obtained from all couples. Normal semen parameters as per WHO guidelines (2010) are sperm concentration >15 million/mL, motility >32%, and morphology >4%.

### 2.2. Optical Tweezers Set-Up

**Figure 1:**
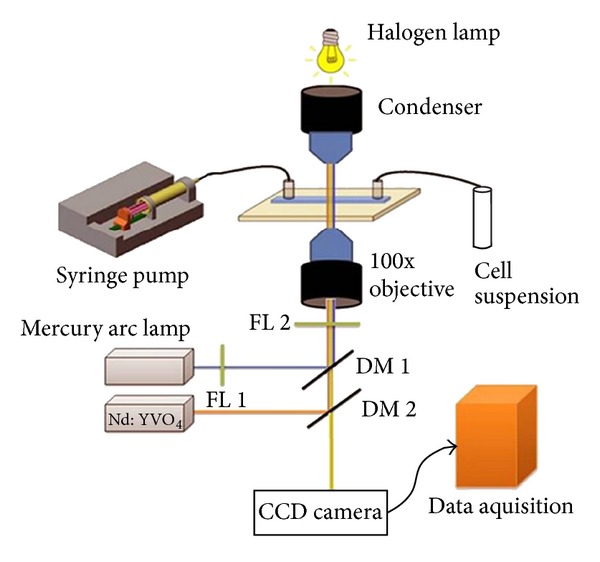
A schematic diagram of optical tweezer set-up used to study rotational dynamics of live spermatozoa.

The optical trap set-up that was used in the present studies has been extensively described earlier [29] and is schematically represented in Figure 1. Briefly, a continuous wave, 1064 laser, beam from a diode-pumped Nd : YVO_4_ laser is coupled to a fluorescence-equipped inverted microscope (NIKON TE 2000U). To achieve trapping, the laser was focused through a 100X microscope objective with numerical aperture 1.3. To avoid possible damage to the trapped sperm cells, the power of the laser incident on the sperm samples was limited to be less than 20 mW. The dynamics of the live sperm cells under the trap were visualized and captured in real-time using a 25-frame/second CCD camera interfaced with a computer. Such videos were then analyzed frame by frame by *Image J *software.

### 2.3. Trapping Efficiency and Rotational Motion

The effect of trapping on the sperm motility is quantified hereafter as the trapping efficiency of our optical tweezers set-up for sperm cells. This was calculated using the following formula [30]:

(1) Trapping  Efficiency  (%) =(1−[pretrap  velocity−posttrap  velocity]pre-trap  velocity)×100.

If the trap has no effect and the velocities before and after trapping remain unchanged, the trapping efficiency is 100%. However any difference between the pretrap and posttrap velocities will cause the efficiency to decrease.

As alluded to previously, upon optical trapping not only the translational motion of the sperm is halted, but also a rotational motion is established. Such motion has been studied in terms of the number of cells that are rotating, their rotational frequency and the beat frequency of the sperm tail through frame by frame analysis of the recorded real-time videos.

### 2.4. Statistical Analysis

Data for each set of cells were analyzed using paired and unpaired *t*-test. Analysis, discrimination, and classification of data were performed with Graphpad prism, Origin, MedCalc, and SPSS software. Statistical significance of the test was defined as *P* < 0.05 and *P* < 0.001.

## 3. Results 

**Figure 2:**
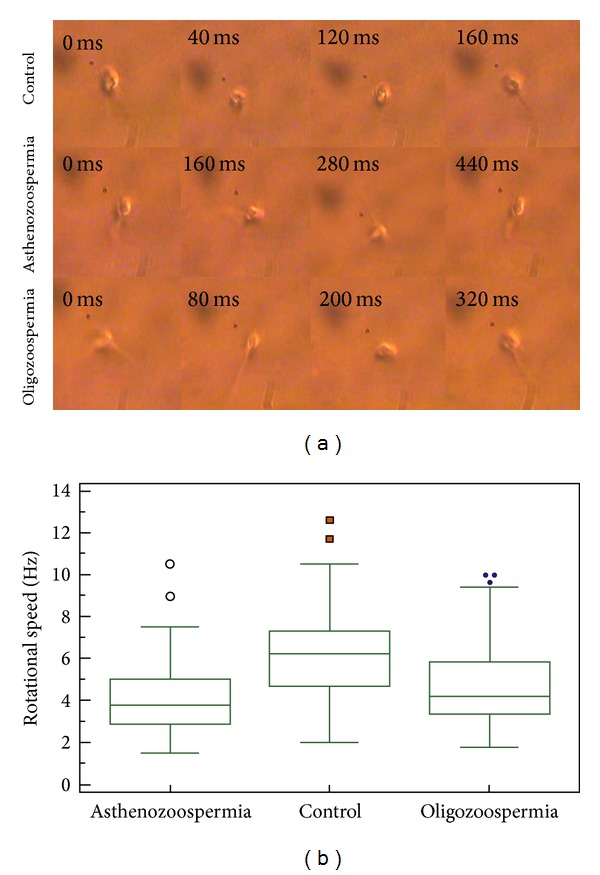
(a) Rotational dynamics of normal and abnormal human sperm cells at different time points on being trapped optically (for a movie clip showing rotational motion of sperm cells under optical trap; see video in Supplementary Materials available online at http://dx.doi.org/10.1155/2014/154367). (b) Box-Whiskers plot represents the rotational speed of normal and abnormal sperm cells compared to each other.

**Figure 3:**
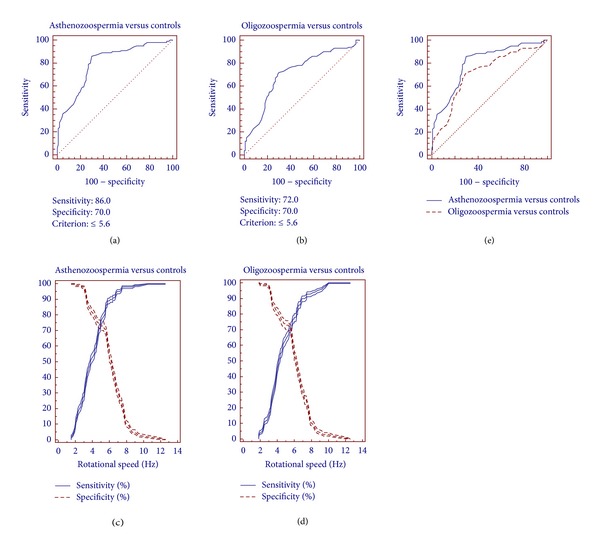
ROC curve analysis indicates cutoff value which has high sensitivity and specificity for discrimination of (a) oligozoospermic sperm cells and controls (AUC = 0.8) (b) asthenozoospermic sperm cells and controls (AUC = 0.714). Sensitivity, specificity, and their 95% confidence of interval of (c) asthenozoospermic and (d) oligozoospermic cases plotted against the sperm rotational speed. (e) Comparison of ROC curves of oligozoospermic and asthenozoospermic cases.

**Figure 4:**
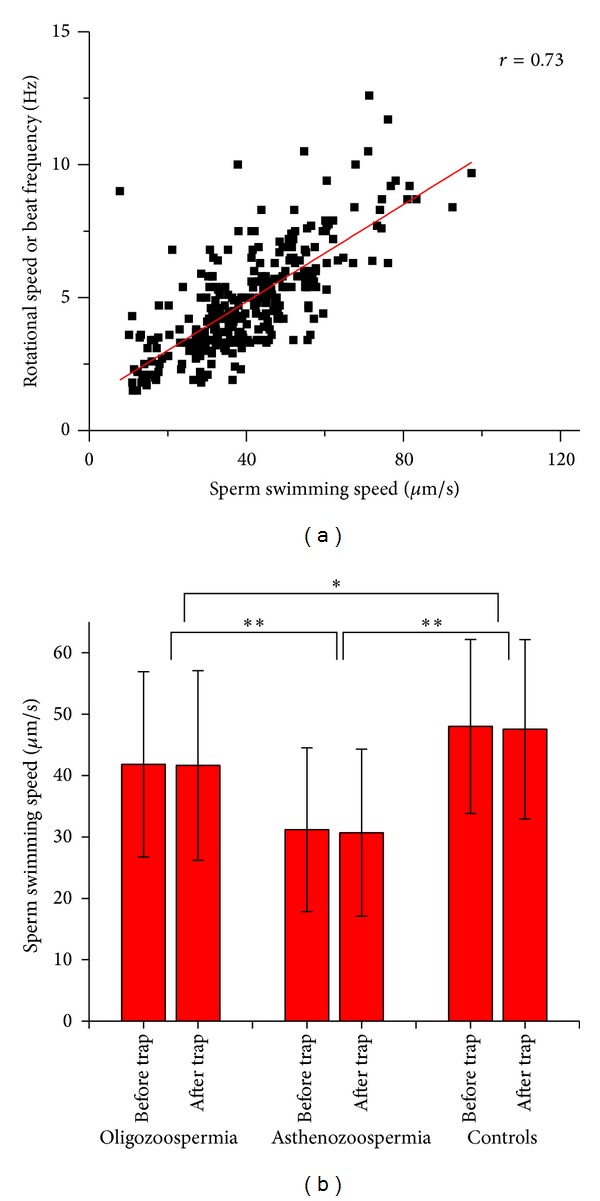
(a) Correlation of sperm swimming speed and rotational speed (beat frequency) of normal and abnormal sperm cells. (b) Measurement and comparison of normal and abnormal spermatozoa swimming speed before and after optical trapping. *P < 0.05, **P < 0.001.

The optical tweezers set-up was used to analyze the rotational dynamics of various categories of sperm cells. As already noted, normal linear translational motion of all motile spermatozoa was halted upon trapping. Cells possessing a functional tail began to rotate under the trap in either a clockwise or anticlockwise direction (Figure 2(a)), with the rotational direction being stochastic. The rotational motion is due to the torque developed by the sperm tail force acting tangentially to the force generated within the optical trap which pins down the head of the sperm (see accompanying video in Supplementary Materials). The rotational speed of oligozoospermic and asthenozoospermic sperm cells was found to be significantly less when compared with controls (Figure 2(b)). Receiver operating characteristic (ROC) curve analysis indicated the beat frequency cut-off value to be 5.6 Hz for differentiating normal and abnormal sperm cells (both oligozoospermic and asthenozoospermic) (Figures 3(a) and 3(b)). Sensitivity and specificity values for differentiating abnormal spermatozoa from normal sperm cells at various beat frequencies are presented in Figures 3(c) and 3(d) for oligozoospermic and asthenozoospermic cases, respectively. The sensitivity and specificity for cut-off value of oligozoospermic and asthenozoospermic cases are 72% and 70% and 86% and 70%, respectively. The rotational speed or beat frequency of various categories of sperm cells also positively correlated (*r* = 0.73) with swimming speed of the sperm (Figure 4(a)). Swimming speeds of oligozoospermic and asthenozoospermic sperm cells were observed to be significantly lower than those of controls (Figure 4(b)). In addition, no significant differences were found between the pretrap and posttrap swimming speeds of oligozoospermic, asthenozoospermic, and normal sperm cells (Figure 4(b)). The trapping efficiency values of the tweezers for oligozoospermic, asthenozoospermic, and normal sperm cells were determined to be 96.2%, 92.2%, and 95.9%, respectively by using formula (1).

## 4. Discussion

Sperm motility is usually evaluated in research and infertility clinics by computer-assisted sperm analysis (CASA) systems which has been commercially available since the mid-1980s [31–33]. Several reports indicate that optical tweezers may be an acceptable alternative for the measurement of sperm swimming force, speed, energetics, and mitochondrial membrane potential [8–10]. Application of this technology not only leads to better understanding of sperm characteristics like motility and energetics, but also directs to inclusive assays for abnormal sperm behavior in infertility and fertilizing ability of sperms.

Upon trapping a sperm cell, we observed conversion of translational motion into rotational motion. We speculate that, following trapping, a reversible change in the molecular components of the mitochondrial apparatus is responsible for the transition from translational to rotational motion. This change gives rise to an enhanced polarizability that, in turn, interacts with the electric field associated with the laser light to generate a torque. The torque that is generated depends on the magnitude of the optical field and, hence, on the laser intensity. Earlier work in our laboratory with live red blood cells has established that rotational motion is dependent on incident laser light intensity (and, hence, on optical field) [34–37].

The sperm, on being optically trapped, rotates at a certain frequency called the beat frequency. This frequency is, in turn, dependent upon the force that is generated by the beating motion of the cell and it correlates well with the sperm swimming speed. The decreased rotational or beat frequency of trapped abnormal sperm cells indicates alteration in the flagellar apparatus or proteins. Further, beat frequency with a cut-off value of 5.6 Hz was found to differentiate oligozoospermic and asthenozoospermic sperm cells from those of controls. However, the area under the curve (AUC) analysis shows that this technique works better for the latter group (AUC = 0.8) than the former group (AUC = 0.714) (Figure 3(e)).

It is necessary to document swimming speed of sperm before and after optical trapping to monitor the sperm physiology before, during, and after optical trapping. Hence, the findings of our present study suggest that the optical tweezers set-up with nIR laser beam does not cause damage to the spermatozoa (Figure 4(b)). This is in good agreement with the findings of König et al. [23] who concluded that “optical micromanipulation of spermatozoa” with long wavelength nIR laser beam causes significantly less damage as compared to ultraviolet exposure. In contrast, long exposure to the laser trap is reported to have an adverse effect on sperm swimming speed and mitochondrial membrane potential due to laser-induced rise in temperature [24, 25]. Also, depolarization of mitochondria has been observed with prolonged exposure of domestic dog spermatozoa to optical tweezers [25].

The “WHO manual for the examination of human semen and sperm (semen-) cervical mucus interaction” (WHO 2010) is considered to be the gold standard for assessing semen quality. An interesting observation worth mentioning is that although, as per WHO guidelines, oligozoospermia refers to deficiency in the number of spermatozoa in semen with motility of the sperm cells remaining normal, beat frequency, an alternative parameter representing sperm motility, was found to be significantly less in oligozoospermia as compared to controls (Figure 2(b)). This is the first time that low motility is seen to be associated with oligozoospermic sperm samples (Figure 4(b)). This observation definitely warrants further investigation. Following validation in a larger sample size, this valuable information can be considered while defining the WHO sperm parameters which, in turn, describe sperm quality.

In conclusion, our work offers indications that optical tweezers are developing into an important tool for optically selecting and sorting sperm cells based on their rotational dynamics and motility. Although further work needs to be undertaken to gain deeper insights into the physics that determines the conversion of a trapped sperm's translational motion into rotational motion, our study shows that a start may be made to utilize this technique to select good quality sperm cells that demonstrate a beat frequency above 5.6 Hz on being optically trapped. Moreover, by studying the pre and posttrap rotational dynamics we have shown that the nIR laser beam of optical tweezers does not damage the normal physiological characteristics of the trapped single live sperm cell. The system provides a multiparametric set of measurements on single live spermatozoa that gives an improved understanding of sperm swimming speed. We suggest that this technique holds considerable potential in selecting good quality sperm cells from infertile men for use in ART procedures such as in-vitro fertilization (IVF) and intra cytoplasmic sperm injection (ICSI), where the sperm quality is a decisive factor in deciding the quality of the resultant embryo.

## Supplementary Material

Real-time video clip showing the rotational dynamics of a trapped sperm cell. The conversion of the translational motion into rotational motion upon trapping a sperm cell is seen.

## References

[1] Ashkin A (1970). Acceleration and trapping of particles by radiation pressure. *Physical Review Letters*.

[2] Ashkin A, Dziedzic JM, Yamane T (1987). Optical trapping and manipulation of single cells using infrared laser beams. *Nature*.

[3] Ashkin A (1998). Forces of a single-beam gradient laser trap on a dielectric sphere in the ray optics regime. *Methods in Cell Biology*.

[4] Bambardekar K, Dharmadhikari AK, Dharmadhikari JA, Mathur D, Sharma S (2008). Measuring erythrocyte deformability with fluorescence, fluid forces, and optical trapping. *Journal of Biomedical Optics*.

[5] Bambardekar K, Dharmadhikari JA, Dharmadhikari AK (2010). Shape anisotropy induces rotations in optically trapped red blood cells. *Journal of Biomedical Optics*.

[6] Ghosh A, Sinha S, Dharmadhikari JA (2006). Euler buckling-induced folding and rotation of red blood cells in an optical trap. *Physical Biology*.

[7] D’Souza JS, Gudipati M, Dharmadhikari JA (2009). Flagella-generated forces reveal gear-type motor in single cells of the green alga, Chlamydomonas reinhardtii. *Biochemical and Biophysical Research Communications*.

[8] Araujo E, Tadir Y, Patrizio P (1994). Relative force of human epididymal sperm. *Fertility and Sterility*.

[9] Shi LZ, Nascimento JM, Chandsawangbhuwana C, Botvinick EL, Berns MW (2008). An automatic system to study sperm motility and energetics. *Biomedical Microdevices*.

[10] Nascimento JL, Botvinick EL, Shi LZ, Durrant B, Berns MW (2006). Analysis of sperm motility using optical tweezers. *Journal of Biomedical Optics*.

[11] Hyun N, Chandsawangbhuwana C, Zhu Q, Shi LZ, Yang-Wong C, Berns MW (2012). Effects of viscosity on sperm motility studied with optical tweezers. *Journal of Biomedical Optics*.

[12] Dantas ZN, Araujo E, Tadir Y, Berns MW, Schell MJ, Stone SC (1995). Effect of freezing on the relative escape force of sperm as measured by a laser optical trap. *Fertility and Sterility*.

[13] Patrizio P, Liu Y, Sonek GJ, Berns MW, Tadir Y (2000). Effect of pentoxifylline on the intrinsic swimming forces of human sperm assessed by optical tweezers. *Journal of Andrology*.

[14] Nascimento JM, Shi LZ, Chandsawangbhuwana C (2008). Use of laser tweezers to analyze sperm motility and mitochondrial membrane potential. *Journal of Biomedical Optics*.

[15] Shao B, Shi LZ, Nascimento JM (2007). High-throughput sorting and analysis of human sperm with a ring-shaped laser trap. *Biomedical Microdevices*.

[16] Garcia MM, Ohta AT, Walsh TJ (2010). A noninvasive, motility independent, sperm sorting method and technology to identify and retrieve individual viable nonmotile sperm for intracytoplasmic sperm injection. *Journal of Urology*.

[17] Tadir Y, Wright WH, Vafa O, Ord T, Asch RH, Berns MW (1989). Micromanipulation of sperm by a laser generated optical trap. *Fertility and Sterility*.

[18] Shi L, Shao B, Chen T, Berns M (2009). Automatic annular laser trapping: a system for high-throughput sperm analysis and sorting. *Journal of Biophotonics*.

[19] Shi LZ, Nascimento J, Chandsawangbhuwana C, Berns MW, Botvinick EL (2006). Real-time automated tracking and trapping system for sperm. *Microscopy Research and Technique*.

[20] Kasai T, Ogawa K, Mizuno K (2002). Relationship between sperm mitochondrial membrane potential, sperm motility, and fertility potential. *Asian Journal of Andrology*.

[21] Nascimento JM, Shi LZ, Meyers S (2008). The use of optical tweezers to study sperm competition and motility in primates. *Journal of the Royal Society Interface*.

[22] König K, Svaasand L, Liu Y (1996). Determination of motility forces of human spermatozoa using an 800 nm optical trap. *Cellular and Molecular Biology (Noisy-le-grand)*.

[23] König K, Tadir Y, Patrizio P, Berns MW, Tromberg BJ (1996). Effects of ultraviolet exposure and near infrared laser tweezers on human spermatozoa. *Human Reproduction*.

[24] Mohanty SK, Sharma M, Gupta PK (2006). Generation of ROS in cells on exposure to CW and pulsed near-infrared laser tweezers. *Photochemical and Photobiological Sciences*.

[25] Mei A, Botvinick E, Berns M Monitoring sperm mitochondrial respiration response in a laser trap using ratiometric fluorescence.

[26] Mohanty SK, Rapp A, Monajembashi S, Gupta PK, Greulich KO (2002). Comet assay measurements of DNA damage in cells by laser microbeams and trapping beams with wavelengths spanning a range of 308 nm to 1064 nm. *Radiation Research*.

[27] Martinez-Pastor F, Johannisson A, Gil J (2004). Use of chromatin stability assay, mitochondrial stain JC-1, and fluorometric assessment of plasma membrane to evaluate frozen-thawed ram semen. *Animal Reproduction Science*.

[28] World Health Organization (2010). *WHO Laboratory Manual for the Examination and Processing of Human Semen*.

[29] Basu H, Dharmadhikari AK, Dharmadhikari JA, Sharma S, Mathur D (2011). Tank treading of optically trapped red blood cells in shear flow. *Biophysical Journal*.

[30] Muthu Rama Krishnan M, Choudhary A, Chakraborty C, Ray AK, Paul RR (2011). Texture based segmentation of epithelial layer from oral histological images. *Micron*.

[31] Aitken RJ, Best FS, Warner P, Templeton A (1984). A prospective study of the relationship between semen quality and fertility in cases of unexplained infertility. *Journal of Andrology*.

[32] Krause W (1995). Computer-assisted semen analysis systems: comparison with routine evaluation and prognostic value in male fertility and assisted reproduction. *Human Reproduction*.

[33] Larsen L, Scheike T, Jensen TK (2000). Computer-assisted semen analysis parameters as predictors for fertility of men from the general population. *Human Reproduction*.

[34] Dharmadhikari JA, Roy S, Dharmadhikari AK, Sharma S, Mathur D (2004). Torque-generating malaria-infected red blood cells in an optical trap. *Optics Express*.

[35] Roy S, Dharmadhikari J, Dharmadhikari A, Mathur D, Sharma S (2007). Study of P. falciparum-infected erythrocytes and induced anisotropies under optical and fluid forces. *Journal of Vector Borne Diseases*.

[36] Zachariah E, Bankapur A, Santhosh C, Valiathan M, Mathur D (2010). Probing oxidative stress in single erythrocytes with Raman Tweezers. *Journal of Photochemistry and Photobiology B*.

[37] Bankapur A, Zachariah E, Chidangil S, Valiathan M, Mathur D (2010). Raman tweezers spectroscopy of live, single red and white blood cells. *PLoS ONE*.

